# Only Delayed

**Published:** 1885-01

**Authors:** 


### ONLY DELAYED.

The continuation of the report of the American Dental Society of Europe, and the report and papers from the Central Dental Association of Northern New Jersey, are crowded out of this number. We regret this, but it was unavoidable.

